# Nanoarchitectonics on living cells

**DOI:** 10.1039/d1ra03424c

**Published:** 2021-05-25

**Authors:** Katsuhiko Ariga, Rawil Fakhrullin

**Affiliations:** WPI Research Center for Materials Nanoarchitectonics (MANA), National Institute for Materials Science (NIMS) 1-1 Namiki Tsukuba Ibaraki 305-0044 Japan ARIGA.Katsuhiko@nims.go.jp; Graduate School of Frontier Sciences, The University of Tokyo 5-1-5 Kashiwanoha Kashiwa Chiba 277-8561 Japan; Institute of Fundamental Medicine and Biology, Kazan Federal University Kreml uramı 18 Kazan 42000 Republic of Tatarstan Russian Federation kazanbio@gmail.com

## Abstract

In this review article, the recent examples of nanoarchitectonics on living cells are briefly explained. Not limited to conventional polymers, functional polymers, biomaterials, nanotubes, nanoparticles (conventional and magnetic ones), various inorganic substances, metal–organic frameworks (MOFs), and other advanced materials have been used as components for nanoarchitectonic decorations for living cells. Despite these artificial processes, the cells can remain active or remain in hibernation without being killed. In most cases, basic functions of the cells are preserved and their resistances against external assaults are much enhanced. The possibilities of nanoarchitectonics on living cells would be high, equal to functional modifications with conventional materials. Living cells can be regarded as highly functionalized objects and have indispensable contributions to future materials nanoarchitectonics.

## Abstract 2

We can introduce functional structures with various components on a living cell as if architectures were constructed on material surfaces.

## Graphical abstract

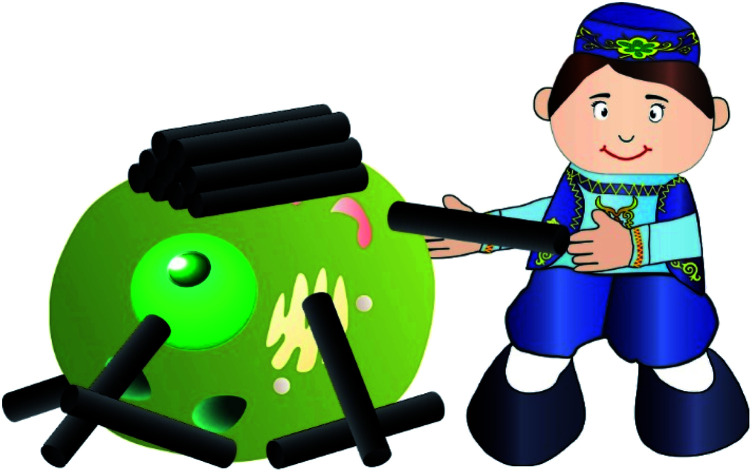

## 1. Introduction

Advancements of our society are supported partially by rapid progress in information technology in cyberspace.^1^ However, the advancements of functional materials in real space are surely necessary in realistic problems such as energy,^2^ environmental,^3^ and biomedical issues.^4^ Chemistry has been playing a crucial role in producing materials with advanced functions, as seen in the continuous progress in organic chemistry,^5^ polymer chemistry,^6^ supramolecular chemistry,^7^ and materials chemistry.^8^ In addition to intrinsic properties of materials, material performances have a significant dependence on their internal structures and organizations.^9^ The latter facts have been revealed through the advanced analyses of nanostructures^10^ and micro/nano-fabrications^11^ as well as in the discovery and progresses on various nanomaterials.^12^ The latter activities are often carried out on the basis of nanotechnology concept.^13^ For further progresses of functional materials for social demands, fusion of nanotechnology with the other fields and methodologies becomes indispensable. It includes organic chemistry, supramolecular chemistry, materials chemistry, micro/nano-fabrications, and bio-related sciences. Such field fusion logically construct functional materials from nano-unit components. This task is assigned to an emerging concept, that is nanoarchitectonics.^14^

**Fig. 1:**
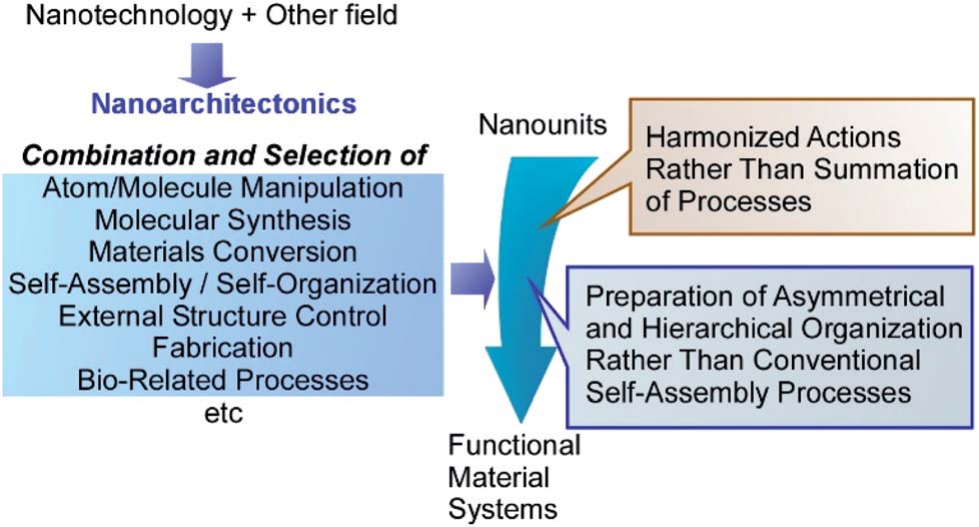
Outline of nanoarchitectonics strategy where functional material systems can be architected from various nano-units through combination and selection of various unit processes including atom/molecule manipulation, molecular synthesis, materials conversion, self-assembly/self-organization, external structure controls, fabrications, and bio-related processes.

Nanoarchitectonics initiated by Masakazu Aono in a conference in 2000 (1st international symposium on nanoarchitectonics using suprainteractions (NASI-1))^15^ similarly to initiation of nanotechnology by Richard Feynman during his lecture entitled “There's Plenty of Room at the Bottom”.^16^ According to the nanoarchitectonics strategy, functional material systems can be architected from various nano-units through combination and selection of various unit processes including atom/molecule manipulation, molecular synthesis, materials conversion, self-assembly/self-organization, external structure controlling, fabrication, and bio-related processes (Fig. 1).^17^ Nanoarchitectonics is not a sole and independent concept, as it has overlaps with pre-existing approaches.^18^ However, it aims to unify the related approaches as a strategy for everything in materials chemistry, similarly to the theory of everything in physics. Therefore, the nanoarchitectonics concept has been employed in a wide range of research fields, such as materials production,^19^ structure formation,^20^ catalyst,^21^ environmental targets,^22^ energy-related applications,^23^ sensors,^24^ and devices.^25^

The nanoarchitectonics processes share some common features with those observed in biology.^26^ Because the nanoarchitectonics processes can include multiple steps for structural construction, nanoarchitectonics is more suitable for preparation of asymmetrical and hierarchical organization^27^ rather than the conventional self-assembly processes based on a simple equilibrium. Biological organizations mostly possess asymmetrical and hierarchical features where the directional energy flow, irreversible materials conversions, and sequential processes become possible with high efficiency and specificity. Some nanoarchitectonics processes rely on the nanoscale interactions, frequently accompanied with uncertainties in nanoscale such as thermal disturbance and probability distributions.^28^ Therefore, the outputs of nanoarchitectonics are the products of harmonized actions rather than a simple summation of all the processes.^29^ The latter situation is also very similar to those occurring in biological systems and functions. Most of biological processes are functioning within the unavoidable thermal fluctuations, where numerous processes are working in certain harmony. Therefore, the nanoarchitectonics concept has been used in biological fields including basic biological systems,^30^ biomedical applications,^31^ and cell-related technology.^32^ It could be said that the final goal of nanoarchitectonics would be the construction of bio-like high functional materials system from common relatively simple nanounits.^33^

**Fig. 2:**
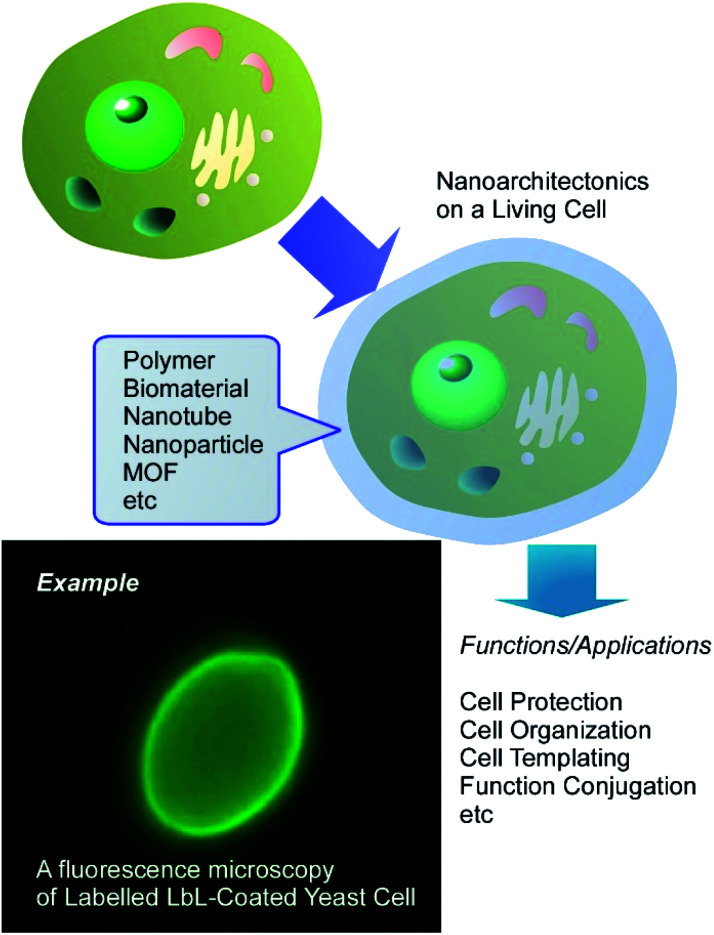
Conceptual representation for cyborg cell prepared through nanoarchitectonics on a living cell.

As the initial step for the final goal, nanoarchitectonics approaches with biological substances including living cells are among the attractive targets. Although routinely other terms rather than nanoarchitectonics are used, such nanostructure formation can be regarded as nanoarchitectonics approach on living cells. For example, Fakhrullin *et al.* proposed a novel concept of cyborg cell for this approach (Fig. 2).^34^ The term “cyborg cells” was devised to denote the cells modified with polymers and/or nanoparticles and thus having their intrinsic biological functions integrated with the functionality of these artificial dopants. A wide variety of living entities can be used as templates for nanoparticle assembly, ranging from unicellular prokaryotes or filamentous fungi to mammalian cells, multicellular cell clusters and whole eukaryotic organisms. The choice of a modification technique is determined mostly by properties of the modified biological system and by a possible need for keeping it viable during and after the modification. Such cyborg cells are already finding applications in creation of bio-electronic devices, biosensors, biosorbents and catalysts, in directed cell delivery, cell-based therapies and tissue engineering. These approaches actually corresponding to nanoarchitectonics on living cells.

Various techniques are widely utilized for the nanoarchitectonics on living cells. For example, the layer-by-layer (LbL) assembly technique has become a very useful tool for creation of functional coatings on the surface of living cells.^35^ LbL surface functionalization comprises the sequential incubation of cells in aqueous solutions of polycations and polyanions, sometimes with inclusion of nanoparticles as an interlayer. An advantage of LbL assembly is that it can be applied to non-uniform areas, such as microorganism surfaces and variable nanoparticulate materials. The duration of the functionalization procedure can be further reduced by using a single-step deposition of polycation-stabilized nanoparticles on the negatively charged cell surfaces. Several methods can be used to test the viability of resulting cyborg cells and organisms, including cell growth monitoring, the use of viability dyes or assessment of biochemical activity of functionalized cells.

In this review article, according to these general background,^15^ the nanoarchitectonics on living cells is firstly explained and discussed. Not limited to conventional polymers, functional polymers, biomaterials, nanotubes, nanoparticles (conventional and magnetic ones), various inorganic substances, metal–organic frameworks (MOFs), and the other advanced items have been used as components for nanoarchitectonics decorations for living cells. These examples encourage us to manipulate and functionalize living substances upon human demands.

## 2. Organic polymer nanoarchitectonics on living cells

The use of polymers for nanoarchitectonics of cell surfaces is somehow a rather basic approach. Coating of cells by organic polymers often becomes essential step for further nanoarchitectonics with the other components. However, various functions can be obtained through a wise selection of the polymer components themselves. Yin, Huang, and co-workers reported protection of living cells from antibiotics in surrounding medium through polymer coating.^36^ In their approach, the surfaces of *Saccharomyces cerevisiae* cells were coated with protective layers upon self-assembly of coacervate microdroplets of carboxyl dextran and carboxymethyl chitosan. This coating nanoarchitectonics did not affect the intrinsic cell viability. The survival rates of the coated cells were significantly improved under the environment contaminated with antibiotics (ciprofloxacin). The coating of the protection layers trapped ciprofloxacin, which suppressed the direct contact of ciprofloxacin with the *S. cerevisiae* cells. The reported cell surface nanoarchitectonics fabricated using biodegradable polysaccharides improves probiotics productivities even in antibiotic environments.

Cell nanoarchitectonics for photothermal-chemotherapy through the combination of intracellular and extracellular modifications of red blood cells was demonstrated by Ma, Yang, and co-workers.^37^ In the initial step, CaCO_3_ nanoparticles were grown upon reaction between Ca^2+^ and CO_3_^2−^ within red blood cells. At the extracellular surface, polymerization of pyrrole resulted in attachment of photothermally active polypyrrole to the cell surface followed by functionalization using folic acid as a targeting reagent. An improved loading of drugs, such as doxorubicin, was obtained due to intracellular CaCO_3_ nanoparticles. The polypyrrole layers led to a light-responsive release of the loaded drugs under near infrared light irradiation, as well as specific targeted functions by formic acids. Multi-task photothermal-chemotherapy was achieved through intracellular/extracellular nanoarchitectonics of living cells.

**Fig. 3:**
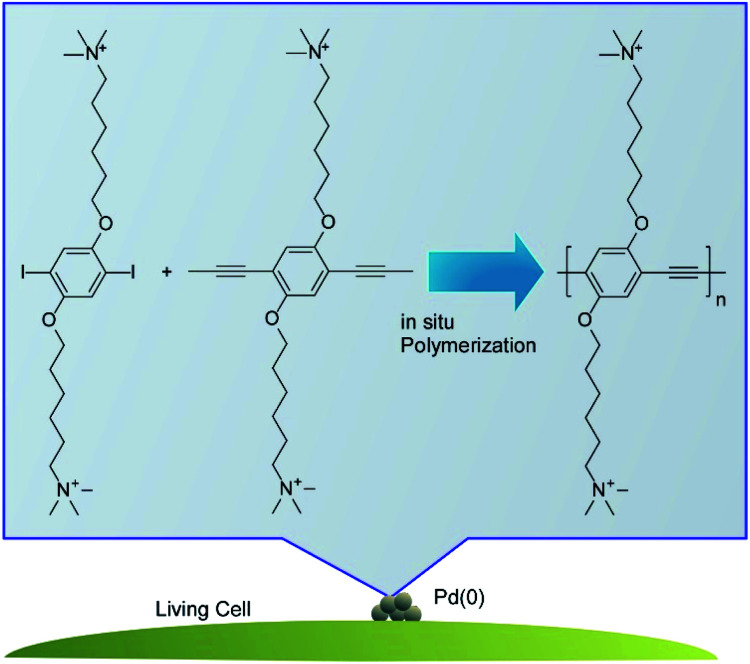
*In situ* synthesis of photoactive polymers on a living cell.

Lv, Wang, and co-workers demonstrated *in situ* synthesis of photoactive polymers on living cells to modulate their biofunctional activities (Fig. 3).^38^ Some functional groups such as carboxyl, phosphate, and amino groups provided Pd binding sites to produce bio-Pd nanoparticles. The Pd nanoparticles often exhibited high activities as catalysts to promote synthesis of photoactive polyphenyleneethynylene through the Sonogashira polymerization on the living cells. In case of *C. pyrenoidosa*, ATP synthesis was enhanced by coating with polyphenyleneethynylene through the improved light absorption. The increase of photosystem I and decrease of photosystem II activities suggest the elevated cyclic electron transport at the membranes of *C. pyrenoidosa* with polyphenyleneethynylene. The regulation in pathways of electron transport resulted in a higher production of ATP.

Cell surface nanoarchitectonics with photothermal cloth on microorganism, *Aspergillus oryzae*, was reported by Wang and co-workers.^39^ Ferric ions were first immobilized onto the surface of *Aspergillus oryzae*, which accelerated *in situ* polymerization of pyrrole to provide polypyrrole as the photothermal cloth on the surface of the microorganism. Photothermal conversion of the photothermal cloth made *Aspergillus oryzae* bioactive in cold environments as confirmed through α-amylase production by *Aspergillus oryzae* and α-amylase catalytic ability. This nanoarchitectonics strategy would be effective for low-temperature environmental adaptation of living cells.

As seen in these example, coating of living cells with organic polymers is useful for introduction of new functions to cells and protection of cells. In addition, polymer coating on cells is essential step for further decoration of living cells with the other components as shown in the following sections.

## 3. Biomacromolecule nanoarchitectonics on living cells

**Fig. 4:**
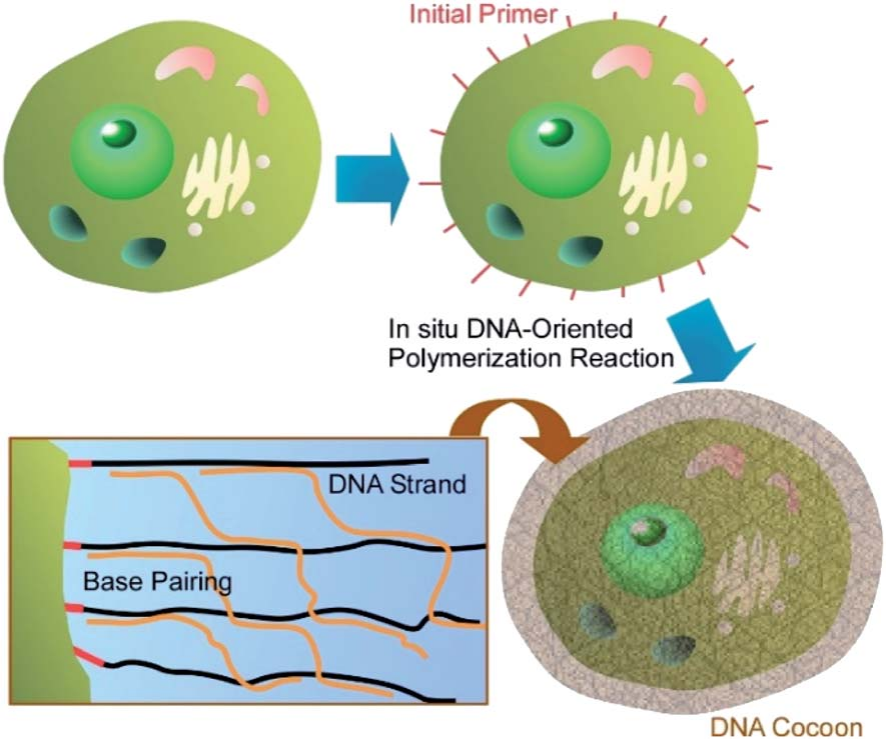
DNA cocoon nanoarchitectonics was supported by *in situ* DNA-oriented polymerization to encapsulate a living cell.

As more functionalized units and more biocompatible components, biomaterials themselves have been used for coating materials on living cells. Li and co-workers proposed a novel cell-surface nanoarchitectonics to fabricate DNA polymer cocoons on living cell surfaces.^40^ As shown in Fig. 4, DNA cocoon nanoarchitectonics was supported by *in situ* DNA-oriented polymerization to encapsulate living cells. Two replication reactions were included in the *in situ* DNA-oriented polymerization. The initial reaction was rimed by initiation primers to induce assemblies of long initial DNA polymers. The following reaction primed by branched primers to result in branched replications. The assemblies of these two kinds of DNA polymers in sequence-specific fashions led to the DNA cocoon structures around the surfaces of living cells. This strategy is applicable for a wide range of cell species including yeast, bacteria, and mammalian cells. In addition, further manipulations such as handling, encoding, and sorting would be included as advanced functions for various biomedical applications.

Wang and co-workers demonstrated the advantages of DNA coating for mammalian cells.^41^ Unlike microbial and plant cells, mammalian cells do not possess any protective exterior cell walls and may suffer damages from environmental assault factors (*e.g.* osmotic pressure, *etc.*). For protection of mammalian cells, they fabricated biomimetic cell walls on the plasma membrane of mammalian cells. Cholesterol-anchored DNA initiators were inserted into the plasma membrane, and then the designed DNA strand and alginate–DNA conjugate formed a supramolecular template upon hybridization chain reaction. The structures formed were cross-linked by polylysine through polyelectrolyte complexation with alginate. High activities of mammalian cells were maintained upon formation of the biomimetic cell walls and their fielding effects were confirmed against both physical and biological assaults. This strategy may be used as a nanoarchitectonics strategy for fabrication of permanent protective covers on mammalian cells.

Choi and co-workers demonstrated a biodegradable modification of living cells using starch polysaccharide as a coating component.^42^ A cationic starch derivative was assembled with an anionic alginate on *S. cerevisiae* cells either in individual state in solution or those on a flat gold surface through LbL assembly technique. The treatment with α-amylase provided opportunities of on-demand degradation of the coated layers in a highly-cytocompatible fashion. Through inclusion of DNA in the LbL shells, a controlled release of DNA upon exposure of the coated cells to an aqueous solution of α-amylase was demonstrated. This nanoarchitectonics strategy would be useful for a certain kind of biomedical applications with some requirements such as programmed *in vivo* dissolution and cytocompatible *in vitro* degradation.

**Fig. 5:**
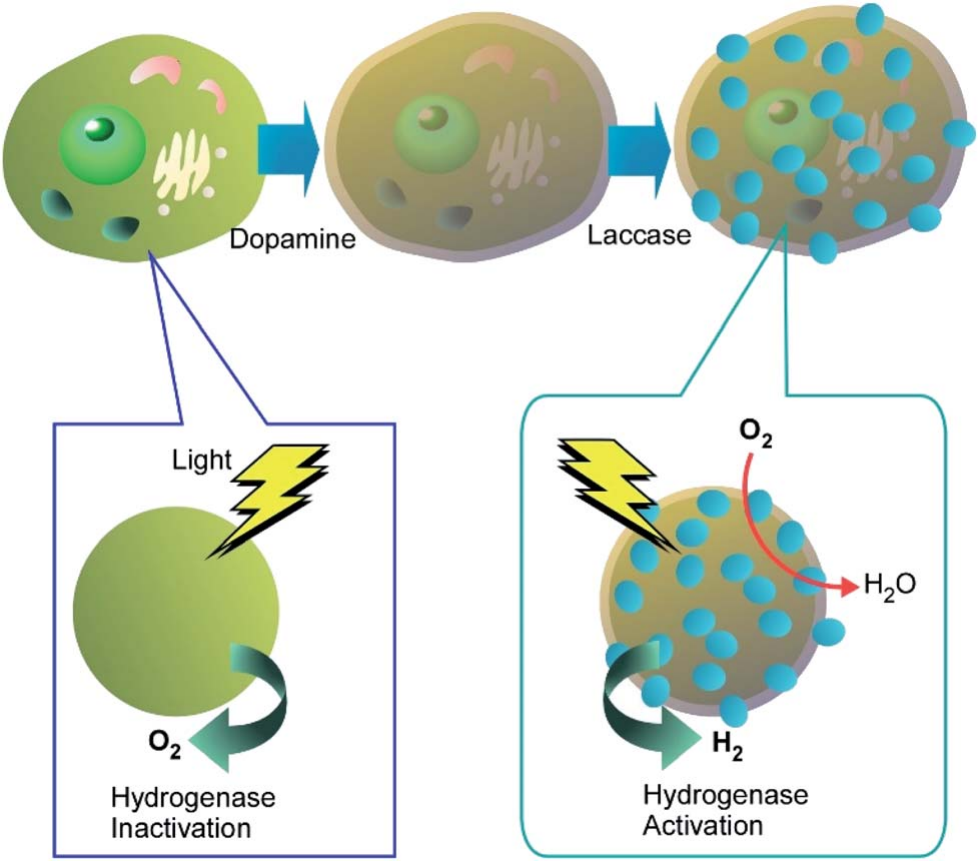
Enzyme-modulated anaerobic nanoarchitectonics on a single cell through sequential assemblies of dopamine, laccase, and tannic acid together with their function of switching from O_2_ to H_2_ production.

Enzyme-modulated anaerobic nanoarchitectonics on a single *C. pyrenoidosa* cell was reported by Huang and co-workers together with their function of switching from O_2_ to H_2_ production.^43^ For this function, sandwich-like enzyme-modulated anaerobic layers were nanoarchitected through the sequential assemblies of dopamine, laccase, and tannic acid on a single *C. pyrenoidosa* cell (Fig. 5). This nanoarchitectonics construction supported an anaerobic balance between O_2_ consumption by mitochondrial respiration and O_2_ production from photosynthesis. Accordingly, the activities of the coated cells were switched from photosynthetic O_2_ production to H_2_ production. This nanoarchitectonics modulation of cell functionalities would be attractive approach for green energy alternatives.

As targeting to cell surface with artificial enzymes, Tanaka and Vong reported their recent approaches to binding artificial metalloenzymes with glycosylation of protein structures on cancer cells.^44^ The artificial metalloenzymes were nanoarchitected through inclusion of metallic biocatalytic sites to the original protein body with the attached glycan-dependent targeting modules. The modification of naturally occurring biomolecules with artificial modules would become exceptionally suitable components of nanoarchitectonics on living cells, where the artificial enzymes using supramolecular structures, biomaterials, and nanomaterials are expected to play unique roles.^45^

Immobilization of functional biomacromolecules on living cells leads to new bio-functions to particular cells. This strategy would play roles on modification of cell functional specialty to target cells. As well as cell protection capability.

## 4. Inorganic nanotube nanoarchitectonics on living cells

Not limited to soft substances, such as polymers and biomaterials with certain shape adaptability, significantly less flexible nanomaterials can also be used as components for cell surface nanoarchitectonics. For example, various one-dimensional nanotubes have been used for modification of cell surface structures.

**Fig. 6:**
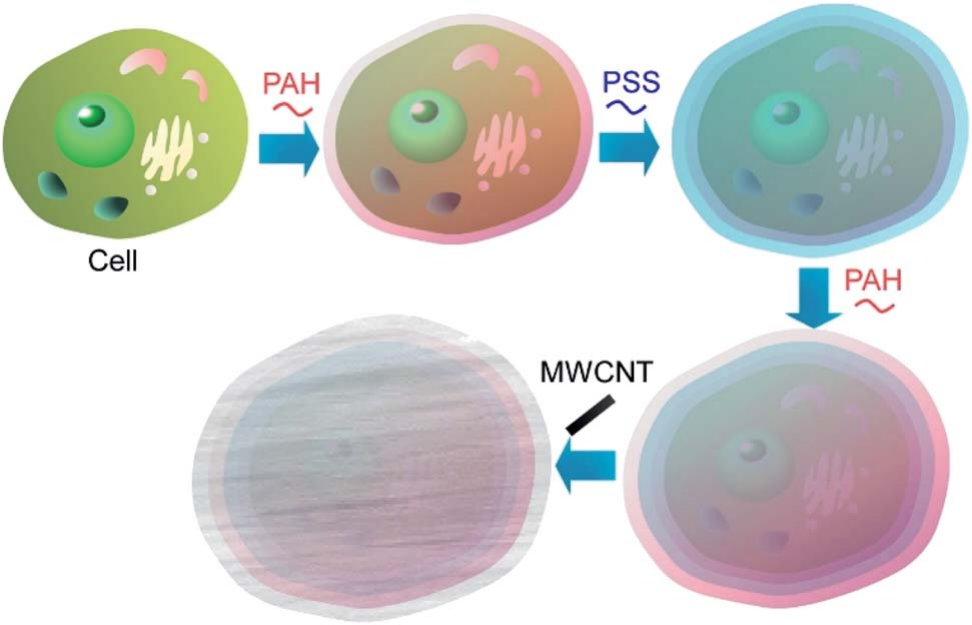
Nanoarchitectonics for composite coatings of polyelectrolytes and oxidized multiwalled carbon nanotubes (MWCNTs) on a living cell.

Living cells can be used as templates for deposition of carbon nanotubes to produce highly organized three-dimensional hybrid microstructures to be applied in bioelectronics and electrochemical biosensors.^46^ The fabrication of composite coatings of polyelectrolytes and oxidized multiwalled carbon nanotubes (MWCNTs) assembled on the cell walls of living *S. cerevisiae* cells was reported (Fig. 6). The oppositely charged polyelectrolytes (cationic poly(allylamine hydrochloride) (PAH) and anionic poly(styrene sulfonate) (PSS)) and MWCNTs were deposited on the individual *S. cerevisiae* yeast cells by the LbL approach with the resulting coating nanoarchitecture of PAH/PSS/PAH/MWCNTs/PAH/PSS. Electrochemical studies using voltammetry and electrochemical impedance measurements demonstrated that the polyelectrolyte/MWCNTs coatings on the yeast cells increased both the signal difference between living and thermally inactivated cells and the accuracy of measurement. Therefore, this method of cells modification with MWCNTs could be a good way to increase the resolution of test systems based on distinguishing between viable and dead cells, such as those applied in toxicity screenings.

The yeast cells encapsulated in a shell of boron nitride nanotubes were proposed as a component of living cell-based biosensors sensitive to hydrophobic environmental pollutants.^47^ Initially, the yeasts were wrapped in polyelectrolyte multilayer coatings and the efficiency of cell modification was controlled by changes in cell Z-potentials. Boron nitride nanotubes functionalized with –OH groups electrostatically attached to the polyelectrolyte-modified yeasts, which was demonstrated by FT-IR as well as various microscopic techniques. The average diameter of the encapsulated yeast cells increased to approximately 5.8 μm as compared to their bare form that was approximately 4 μm. Importantly, the physiological activity in the cells was not changed as result of the cell modification.

**Fig. 7:**
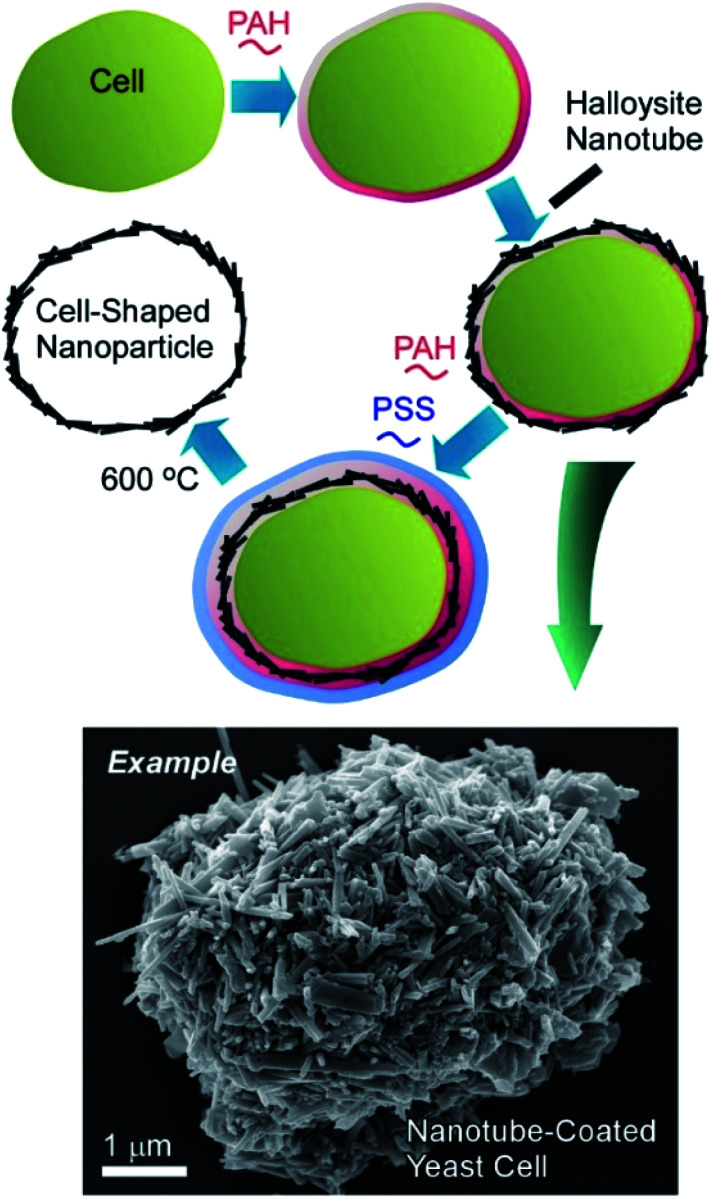
Nanoarchitectonics with halloysites (clay nanotubes) on a living cell with an example image.

Naturally occurring nanotubes, such as halloysites (clay nanotubes), have been recently paid attention.^48^ Halloysite nanotubes have been used as active components of nanoarchitectonics on living cells (Fig. 7).^49^ The LbL assembly of clay nanotubes on living yeast cells followed by the thermal decomposition of the cell scaffolds allows for fabrication of hollow three-dimensional biocompatible ceramic microcapsules useful for delivery and sustained release of chemical inhibitors and drugs. Yeast cells were first coated with a layer of cationic (poly)allylamine hydrochloride (PAH) to facilitate the attachment of the negatively charged halloysite nanotubes. Another bilayer of PAH/PSS was used as glue to secure the halloysite nanotubes layer on the yeast cell walls. The hollow capsules obtained after the thermal decomposition of yeasts at 600 °C reproduced the original ellipsoidal shape of the native yeast cells. Without thermal treatment, viable artificial hybrid inorganic-cellular structures (“armoured” cells) were generated. The budding of the modified cells was delayed with appearance of “bare” daughter cells not bearing the nanotube–polyelectrolyte shell, suggesting the use of the clay encapsulation of microorganisms as a method of controlling cell growth.

The related approaches were further expanded to use the functionalized halloysite nanoclay. The lumen of the halloysite nanotubes can be loaded with various substances to provide nutrition for the encapsulated cell. Additionally, the clay nanotubes used in artificial cell coatings can be modified with metal oxide nanoparticles to impart the cell with magnetic properties.^50^ To modify the clay nanotubes with Fe oxide nanoparticles, the composites were directly synthesized in the presence of the nanotubes. As a result, magnetic clay nanotubes were obtained with Fe oxide nanoparticles located both in the lumen and on the outer nanotube surface. These magnetic nanotubes were slightly positive, allowing their direct deposition onto the viable yeasts. The modification of yeast cells with 200–300 nm thick shell of magnetic nanotubes did not affect the viability or vitality of yeast, but allowed magnetic-field manipulation of the coated cells and their separation.

**Fig. 8:**
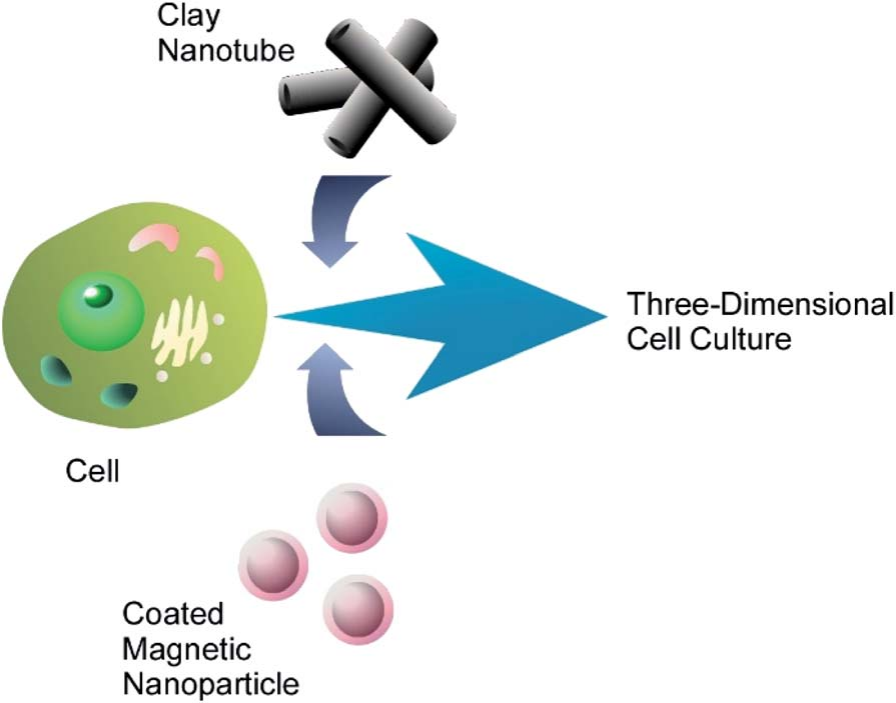
Nanoarchitectonics modification of a living cell with magnetic nanoparticles and clay nanotubes for fabrication of three-dimensional cell cluster.

The magnetic mammalian cells can be grown not only as mono- or multilayer cell cultures, but also assembled in three-dimensional clusters, or spheroids, which have now become an important tool in cancer research due their morphological and physiological resemblance to solid tumours. A cluster of magnetic cells retains the magnetic properties of constituent cells and can be displaced as a whole using an external magnetic field. The formation of multicellular spheroids consisting of one or two types of magnetically modified cells was demonstrated.^51^ Additionally, the cells were modified with clay nanotubes for extended functionality (Fig. 8), and such a modification did not interfere with the magnetic properties of the resulting three-dimensional cell cluster. Three-dimensional cell cultures (especially the mixed ones) are often used in drug research and testing because they are more effective as substitutes for natural tissues than regularly-used cell monolayers.

Although hairs in mammals are not cells, these filaments are generated by cells and also possess biological microstructure. Halloysite nanotubes can self-assemble on the surfaces of human or animal hair, forming a robust multilayer coating held non-covalently on the hair surface and stabilized by inter-tube van der Waals forces.^52^ The spontaneous assembly of the nanotubes begins in the hair cuticle and the layer of the nanotubes becomes thicker as it dries. The density and thickness of the clay coating can be controlled by optimizing the suspension concentration, pH and halloysite hydrophobicity. The coating preserves stability even after 1 h of washing in swirling water or shampoo, justifying the use of on-hair halloysite self-assembly as a means of delivering various therapeutic preparations or colouring agents to the hair surface. The release of the loaded drug can be regulated through modification of the nanotubes with natural or synthetic polymers.

**Fig. 9:**
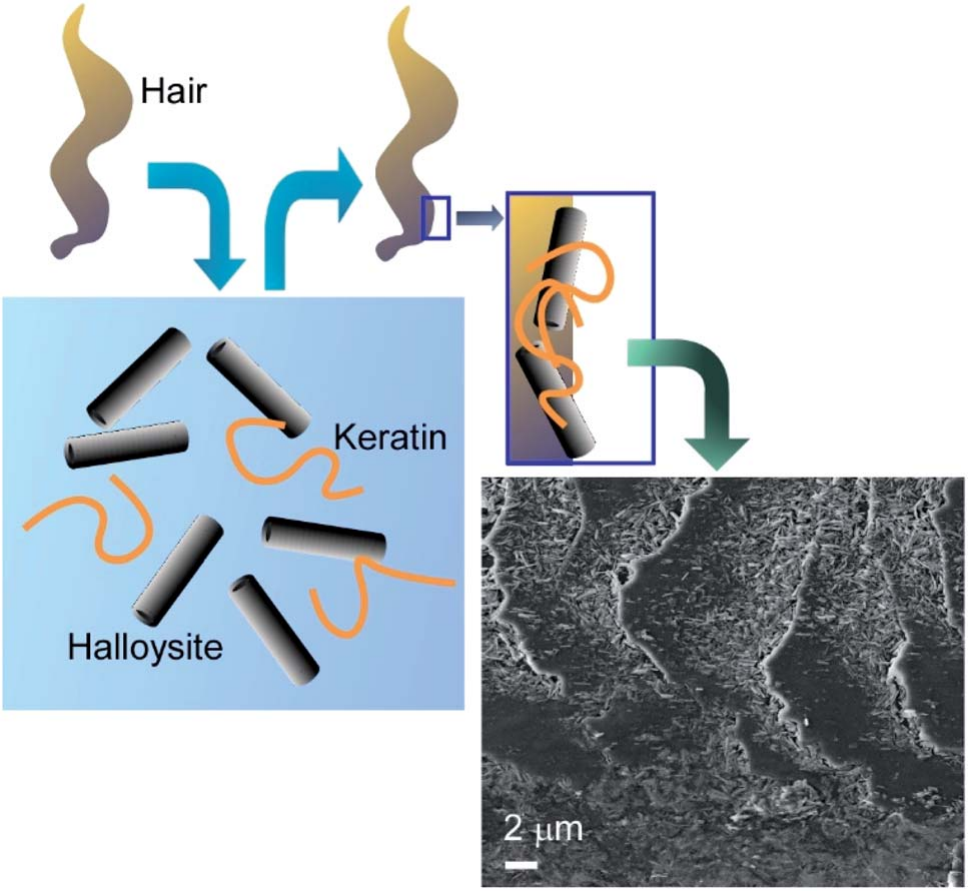
UV-protective coating of based on human hair with keratin and natural halloysite clay nanotubes (surface SEM image is attached).

The obvious primary goal of creating artificial coatings on hair or living cells is the protection of the underlying biological objects against adverse environmental conditions. A novel UV-protective coating based on keratin and natural halloysite clay nanotubes was proposed for surface engineering of human hair (Fig. 9).^53^ The hair surface coverage reached 50–60% after 1 h of hair immersion in 1 wt% halloysite/keratin dispersion. The composite material built from the hydrolysed keratin and halloysite nanoclay demonstrated excellent adhesion to natural keratin of hair cuticle. Halloysite-enhanced keratin binding to the hair surface provided a long-term repairing action. The hair coating efficiency correlated to the UV protective action of the composite, which was supported by studying hair morphology with atomic force microscopy (AFM) and dark-field hyperspectral microscopy. The halloysite/keratin mixture exhibited a better protection for the hair structure compared to pure keratin suspension after 24, 48 and 72 h exposure to UV light.

A protective capsule of clay nanotubes allows for the storage of bacterial cultures at room temperature in the form of liquid marbles.^54^ The liquid marble is a structure reversal to an oil-in-water Pickering emulsion and consists of a water core stabilised by hydrophobic particles. Clay nanotubes rendered hydrophobic through silane grafting of long alkane groups were used to encapsulate a suspension of bacteria in a nutrient enriched medium. The bacteria could survive, proliferate, and produce a biofilm inside the core of the liquid marble in air without hindrance from the clay shell. Each marble initially contained about 10^3^ microorganisms but after 10–20 h the bacteria multiplied their numbers forming a biofilm located at the inner surface of the halloysite shell. The formation of the biofilm further reinforced the capsule and reduced the evaporation of the aqueous medium. All tested microbial species (*A. borkumensis*, *E. coli* and *S. cerevisiae*) could be stored for at least 4 days in dried nanoclay capsules at room temperature without loss of viability. This technique of a robust encapsulation of a relatively large numbers of microorganisms can find numerous applications, for instance, in oil spill remediation, wastewater treatment or probiotic delivery.

Although inorganic nanotubes such as carbon nanotubes and clay nanotubes are not always biocompatible components, coating of living cells with these inorganic nanotubes become possible with the aid of polymers and LbL assembling technique. These cell decorations are useful for introduction of inorganic-material-specified functions to living cells.

## 5. Nanoparticle nanoarchitectonics on living cells

Zero-dimensional nanomaterials, nanoparticles, are frequently used as functional components for nanoarchitectonics on living cells. In particular, cells decorated with magnetic nanoparticles have various possibilities in applications because manipulatable features are introduced. In the following sections, the recent examples of particle-modified cells are classified into magnetic ones and others.

### 5.1. Various nanoparticles

**Fig. 10:**
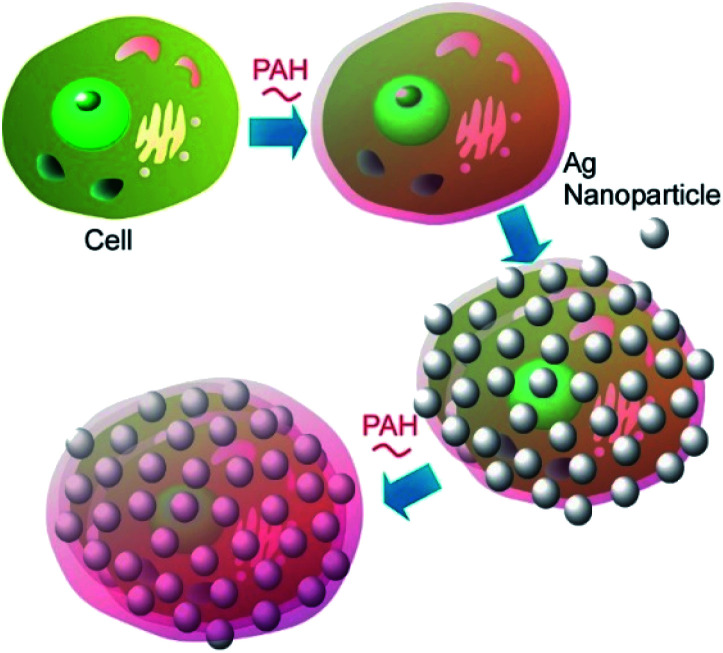
Coating of a cell with polyelectrolyte shells containing noble metal nanoparticles such as Au nanoparticles and Ag nanoparticles.

Coating the bacterial cells with polyelectrolyte shells containing noble metal nanoparticles (Au nanoparticles and Ag nanoparticles) can improve the identification and characterization of bacteria by the surface-enhanced Raman scattering (SERS) technique (Fig. 10).^55^ The negatively charged Ag nanoparticles and Au nanoparticles were attached to the surface of *Escherichia coli* and *Staphylococcus cohnii*, the Gram-negative and Gram-positive bacteria, respectively, through an interlayer of positively charged PAH molecules. Another layer of PAH was deposited to prevent the occasional detachment of nanoparticles due to mechanical impacts. Both isolated and aggregated nanoparticles were present on the bacterial cell wall as was evidenced by the SEM and AFM studies. The modification of bacterial cells with noble metal nanoparticles facilitated the visualization of bacteria under regular white-light microscope and identification of a single bacterial cell with SERS. The important information about the cell wall biochemistry and its interaction with noble metal nanoparticles could also be obtained during analysis of SERS spectra of the modified bacterial cells.

**Fig. 11:**
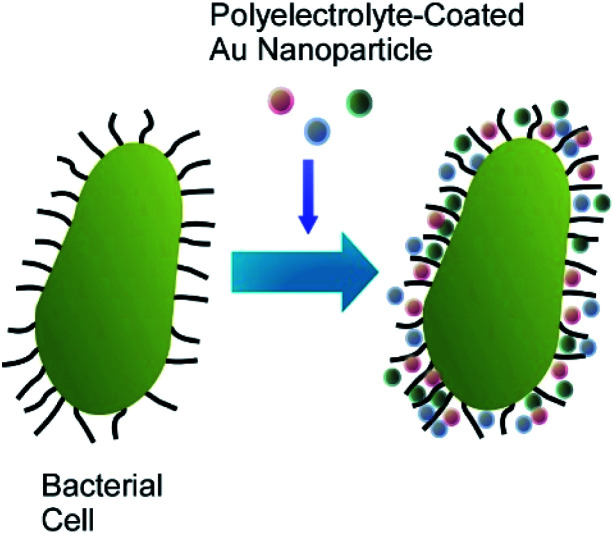
Fabrication of Ag nanoparticle-modified a bacterial cell with the aid of three kinds of cationic polyelectrolytes.

A detailed study of the fate of swallowed “nanobaits” inside the *C. elegans* intestines was performed using Ag nanoparticles.^56^ PAH-modified Ag nanoparticles attached to living bacterial cells were delivered to the nematodes. The enhanced dark field microscopy study demonstrated that once the carrier bacterial cells were grinded and digested inside the nematode digestive tract, most of the attached nanoparticles were released and could potentially travel freely in the organism. A more straightforward way to fabrication of Ag nanoparticle-modified bacterial and yeast cells was also tested. Three cationic polyelectrolytes, (poly(allylamine) hydrochloride (PAH), poly(diallyldimethylammonium) chloride (PDDA) and poly(ethyleneimine) (PEI)) were used as stabilizers for citrate-capped silver nanoparticles (Fig. 11). After stabilization, the now positive nanoparticles directly attached to microbes, resulting in fabrication of uniform nanoparticle coatings, similar to those obtained using the conventional LbL approach.

Chirality controls in artificial material systems have been paid much attention.^57^ However, chirality is not usually created with conventional materials synthesis. Chirality transfer from naturally occurring biosystems to artificial materials are often effectively utilized. For example, Kotov and co-workers demonstrated chiral plasmon activities through immobilization of plasmonic Au nanoparticles on a helical bacterium, *Campylobacter jejuni*, which possesses right-handed helical morphologies with a pitch of 1–2 μm.^58^ Chiroptical activity at plasmonic regions (500–750 nm) was confirmed in the nanoparticle-coated bacteria cells, although the bacteria themselves did not show any rotatory activities. Simulations have confirmed that the observed chiroptical activity was originated in helical assembly of gold nanoparticles. This work provides insights to use chiroplasmonic particles nanoarchitected on living cells for various applications such as bioanalysis.

**Fig. 12:**
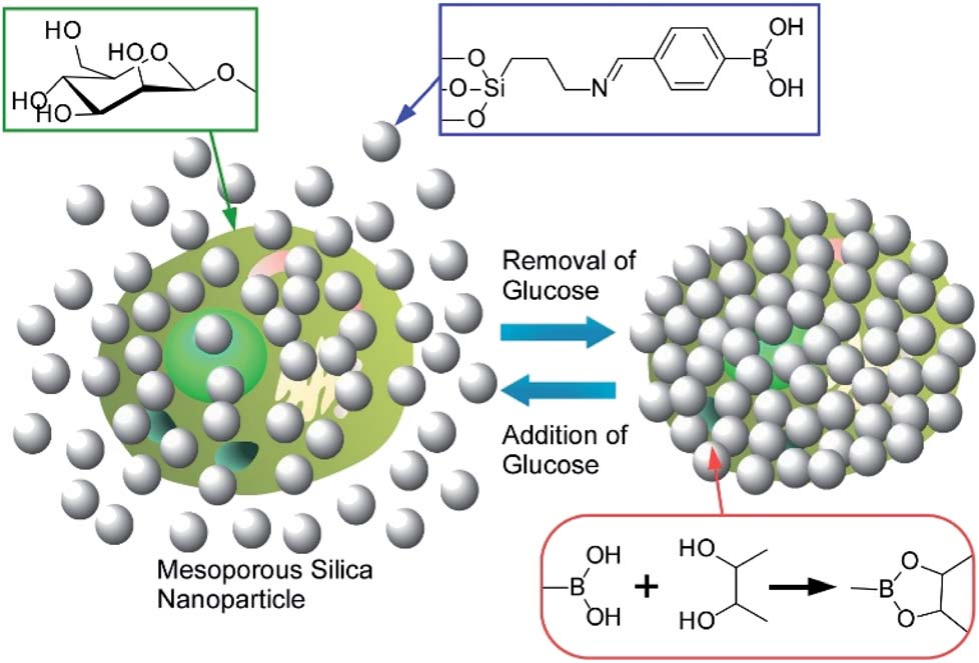
Reversible coating of a living cell with mesoporous silica nanoparticles on the basis of click reaction between boronate B(OH)_2_ group on the particles and polysaccharide residues at surfaces of the cell.

Yang, Su, and co-corkers reported a nanoarchitectonics method for a living cells modification on the basis of click reactions.^59^ The proposed method leads to reversible encapsulation of single yeast cells by mesoporous silica nanoparticles (Fig. 12). The used mesoporous silica nanoparticles were grafted with boronate B(OH)_2_ group that can be reversibly reacted with polysaccharide residues at cell walls of yeast cells. The formed connection can be reversibly broken and re-formed by addition and removal of glucose, respectively. This cell surface nanoarchitectonics led to effective protection of living cells under harsh conditions as well as reversible of abiotic functions. Important point is the absence of necessities of modification of target cells themselves. Therefore, the reversible regulations of cell coating can be applied over several daughter cell generations. It would be a highly versatile method for reversibly controllable cell protection and bio-functions with single living cells for the next generation biotechnology.

### 5.2. Magnetic nanoparticles

Functionalization of living cells with magnetic particles to produce viable “magnetic” cells is important for toxicity and genotoxicity screening applications. Several approaches have been investigated for the fabrication of magnetic yeast cells though polyelectrolyte mediated deposition of superparamagnetic iron oxide nanoparticles on the surface of living yeast cells.^60^ Negatively charged rod-like magnetic nanoparticles stabilized with metal–tetramethyl ammonium hydroxide or positively charged PAH-stabilized spherical magnetic nanoparticles were immobilized on the surface of polyelectrolyte-coated yeast cells. Alternatively, a direct single step ‘‘magnetization’’ of yeast cells was performed by deposition of the PAH-modified nanoparticles on native yeasts. TEM observations demonstrated the formation of a relatively thick (80–130 nm) and uniform layer of magnetic nanoparticles on the cell surface. The modified cells remained viable and could be manipulated and positioned with an external magnetic field. The ‘‘magnetization’’ of yeast cells expressing green fluorescent protein (GFP) under the control of RAD54 promoter allowed obtaining a controllable living sensor responsive to DNA damage by toxic compound.

**Fig. 13:**
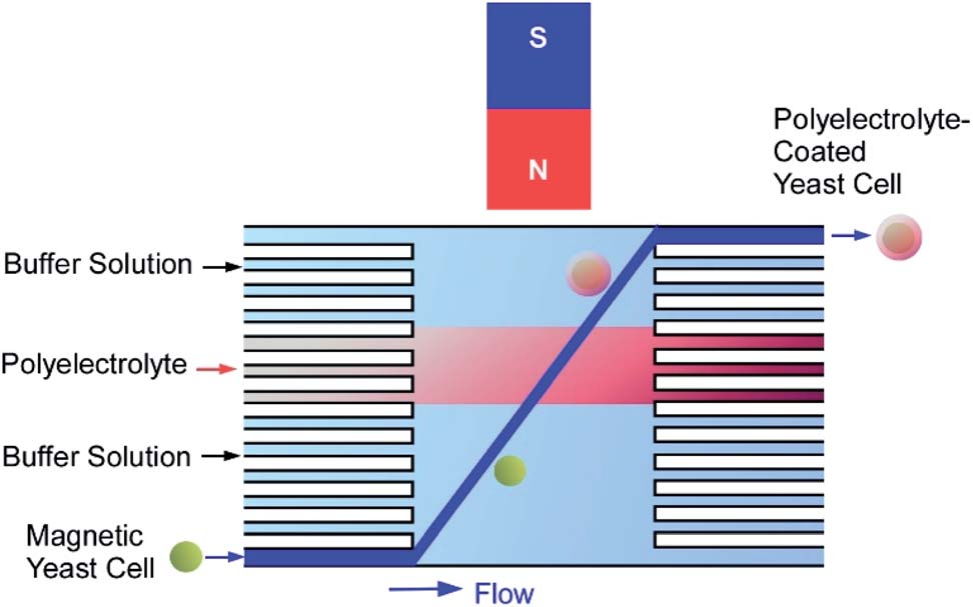
A microfluidic device designed for fast and automated deposition of materials onto magnetically functionalized yeast cells.

The manual fabrication of cyborg cells (surface-nanoarchitected cells) by LbL technique is a rather laborious and time-consuming process because of many deposition and washing steps. To overcome this problem, a microfluidic device was designed for fast and automated deposition of materials onto magnetically functionalized biotemplates (Fig. 13).^61^ Living yeast cells functionalized with magnetic nanoparticles were introduced into a microfluidic chamber and moved across the sequential co-laminar streams of polyelectrolyte and washing buffers by the action of a magnetic field, allowing the coating and washing processes to be condensed into a single step. The whole process took less than 90 s and could be visualized when the negatively charged magnetic yeast cells passed through the stream of fluorescently labelled cationic polyelectrolyte. The development of this automated procedure opens the possibility of industrial production of genotoxicity and cytotoxicity biosensors or bio-templated hollow microcapsules.

A simple and low-cost toxicity screening bioassay method was developed on the basis of viable magnetic green fluorescent protein (GFP) reporter yeast cells held within a microfluidic device by the external magnetic field.^62^ “Glass-on-glass” and “polydimethylsiloxane (PDMS)-on-glass” microchips were designed and introduced into microfluidic devices, capable of generating concentration gradients of tested substances and containing magnetically functionalized GFP reporter yeast cells. Yeast cells were obtained by modification with PAH-stabilized magnetic nanoparticles and retained in the device by simply placing a small neodymium magnet on the chamber top. A statistically significant and dose-dependent increase in fluorescence was observed upon exposure of the magnetic GFP reporter yeasts to increasing concentration of a genotoxic compound. After the magnet withdrawal the yeast could be easily flushed out from the devices. The facile repeated removal and reloading of the yeast cells allows easy automatization of the whole assay.

Artificially obtained magnetic bacteria of selected species can be used as a bioremediation means to address environmental pollution.^63^ The surface of hydrocarbon-degrading Gram-negative marine bacteria *Alcanivorax borkumensis* was modified with polycation-coated iron oxide nanoparticles in a single step deposition process. PAH-stabilized magnetic nanoparticles did not affect the viability, growth and proliferation of the marine bacteria. The magnetic coating was removed after several cell divisions providing generations of native, non-modified cells. The presence of magnetic nanoparticles did not interfere with the ability of the cells to colonize hydrophobic substrates in the form of biofilms, as well as their biosurfactant production. At the same time, it provided the opportunity of magnetic manipulation over these hydrocarbon-degrading microorganisms, facilitating their use for the elimination of marine oil spills.

The functionalization of microorganisms with nanoparticles can be used as a way to more uniform and controllable delivery of nanoparticles to higher organisms in order to assess the toxicity of nanoparticles and their transfer along the food chains.^64^ Bacterial (*E. coli*) and microalgae (*C. pyrenoidosa*) cells were coated with nanoparticles *via* the direct deposition of polycation modified magnetic nanoparticles or *via* the LbL assembly of polyelectrolyte multilayers doped with anionic Ag nanoparticles. Such nanoparticles-modified microbes played the role of “nanobaits”, swallowed by *C. elegans* nematodes during their normal feeding. The internalized nanoparticles were observed exclusively in the digestive tract of the transparent worms. The swallowing of iron oxide nanoparticles rendered the living nematodes magnetically-responsive, allowing the magnetic manipulation and separation of the nematodes from the medium.

**Fig. 14:**
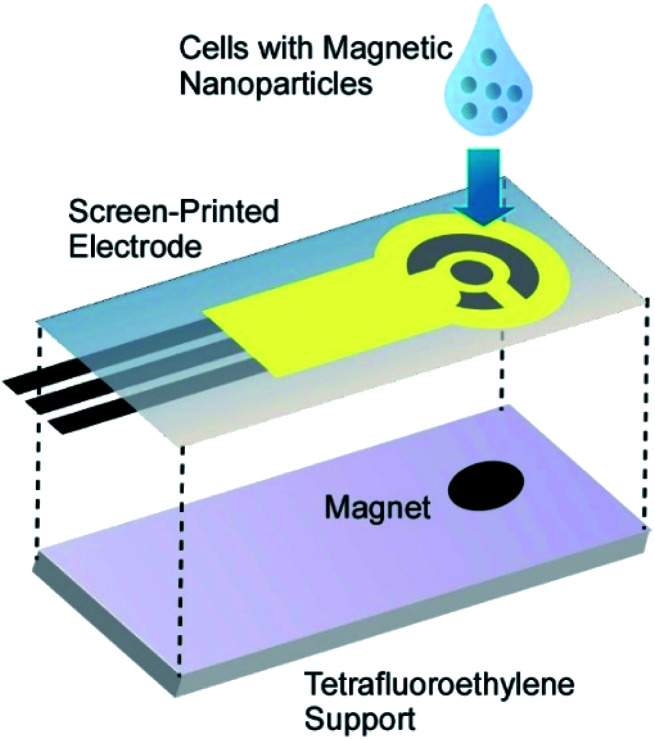
The biosensor consisted of cells coated with PAH-modified magnetic nanoparticles on the surface of an electrochemical screen-printed electrode with a permanent magnet.

Viable magnetic cells of green photosynthetic unicellular microalgae *C. pyrenoidosa* were utilized as a component in a novel biosensor for quantitative assessment of environmental contaminants.^65^ Various pollutants act as inhibitors of the microalgae photosystem II (PSII), allowing development of microalgae-based express diagnostic tools to rapidly assess the quality of drinking water. The biosensor consisted of *C. pyrenoidosa* cells coated with PAH-modified magnetic nanoparticles and retained on the surface of an electrochemical screen-printed electrode with a permanent magnet (Fig. 14). As a proof-of-principle, the applicability of the developed biosensor to the fast detection of two triazine herbicides, atrazine and propazine was demonstrated. The current of ferricyanide ion was recorded during alternating illumination periods. The biosensor was capable of detecting atrazine (from 0.9 to 74 mM) and propazine (from 0.6 to 120 mM) with the detection limits of 0.7 and 0.4 mM, respectively.

A detailed toxicity assay of PAH-modified magnetic nanoparticles to primary and cancer human cells was provided.^66^ The viability staining using an ethidium bromide/acridine orange dye mixture demonstrated that magnetic functionalization did not affect the integrity of cell membranes in magnetic nanoparticles-coated human skin fibroblasts and adenocarcinomic alveolar epithelial cells A549 cells. The presence of magnetic nanoparticles on the cell surface did not induce apoptosis in cancerous cells (A549) and caused a negligible level of apoptosis in primary (HSF) cells. No changes in lysosomal activity or F-actin distribution in magnetically modified cells were found. The magnetic nanoparticles formed a mesoporous coating on the cell surface, which did not interfere with the cell adhesion to the substrate, spreading and proliferation. However, after 24 h of growth some magnetic nanoparticles were found inside the cells as evidenced by TEM imaging, suggesting that magnetic nanoparticles derived from the coating could be partly internalized and distributed between daughter cells during proliferation.

**Fig. 15:**
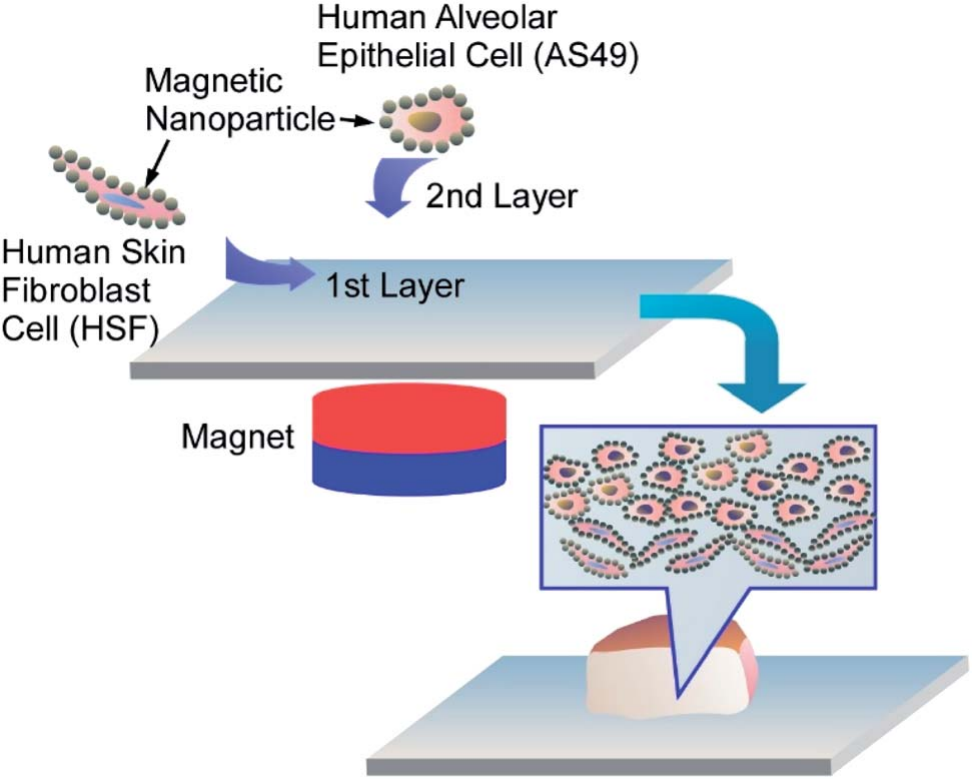
Nanoarchitectonics for layered multicellular clusters mimicking the real human tissues using adenocarcinomic human alveolar epithelial cells (A549) and human skin fibroblasts (HSF) coated with PAH-magnetic nanoparticles.

The direct one-step surface modification of human cells with polyelectrolyte-stabilized magnetic nanoparticles allows magnetically facilitated scaffold-free assembly of two-layered multicellular clusters mimicking the real human tissues (Fig. 15).^67^ The positively-charged PAH-magnetic nanoparticles self-assembled on negatively-charged A549 and human skin fibroblasts, resulting in formation of the uniform magnetic nanoparticles monolayer on both A549 cells and fibroblasts. Using a permanent magnet, the first layer of the magnetic nanoparticles-coated HSF cells was assembled in a culture plate, and then a layer of magnetic nanoparticles-coated A549 cells was added. After the magnet removal the cells were able to form lung-tissue mimicking porous clusters. Both A549 and HSF cells not only remained viable after magnetic functionalization, but demonstrated the higher proliferation rate when compared to intact cells. Once the magnets were removed, the multicellular film could be easily detached from the well, but magnetic nanoparticles retained between the cells preserved the option of magnetic manipulation with the clusters.

Various nanoparticles can be also immobilized on to living cells. As shown in various examples in nanoarchitectonics of cells with magnetic nanoparticles, functions far from natural cells can be introduced. Because functions of nanoparticles are really rich, nanoparticle-based cell-surface nanoarchitectonics would create lots of possibilities in applications.

## 6. Other inorganic nanoarchitectonics on living cells

Various other inorganic substances have been used for cell-surface nanoarchitectonics. For example, the development of hybrid cellular inorganic core–shell microparticles by the encapsulation of individual living yeast cells *S. cerevisiae* in calcium carbonate microshells was reported.^68^ The inorganic shells were obtained by direct precipitation of CaCO_3_ from supersaturated solutions of Ca^2+^ and CO_3_^2−^ ions on the cell walls of living yeast cell templates. A uniform layer of crystalline calcium carbonate formed on the cell surface was birefringent when observed in polarized light and had a crystalline structure similar to vaterite and partly calcite according to X-ray powder diffraction (XRD) analysis. The micrometer-thick calcium carbonate shells did not prevent the transfer of low-molecular weight substances into the encapsulated cells and the cells remained viable even after several weeks of storage. This method can be regarded as an artificial approach mimicking the biomineralization process occurring in nature in unicellular microorganisms, where it functions to protect the cells from harsh environmental conditions.

**Fig. 16:**
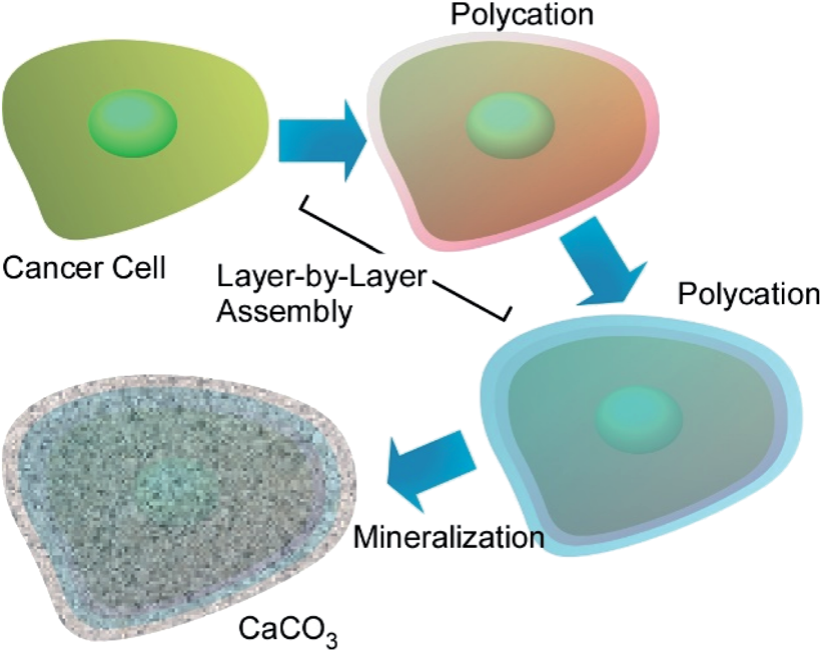
Cancer cell-surface nanoarchitectonics with polyelectrolytes *via* LbL assembly followed by calcium carbonate (CaCO_3_) coating upon biomineralization.

Huang and co-workers investigate suppression of cancer cell migration upon cell-surface nanoarchitectonics.^69^ In their approach, SKOV-3 and HeLa cells were coated with polyelectrolytes *via* LbL assembly followed by calcium carbonate (CaCO_3_) coating upon biomineralization (Fig. 16). These shell structures, especially mineral shell, effectively suppressed migration of SKOV-3 and HeLa cells even though any anticancer drugs were not added. MMP-9 secreted by cancer cells was significantly increased after the cells were coated with these films and minerals, which suggests certain relation of MMP signalling pathways with cell migration inhibition. These mineral nanoarchitectonics on cancer cell surface would become drug-free strategy for suppression of the motility of cancer cells.

**Fig. 17:**
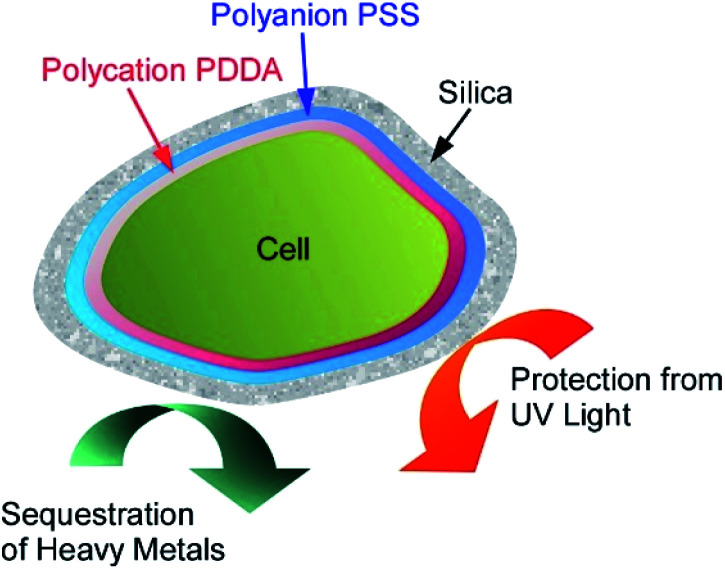
Protection of a loving cell with cationic PDDA and anionic PSS and additional coating with silica shells.

For the use of living cells for unusual applications such as removal of heavy metals from industrial wastewater, stability of living cells has to be improved. Wang, Su, and co-workers reported protection of cyanobacterial strains of the genus *Synechococcus* sp. PCC7002 through LbL covering with cationic PDDA and anionic PSS that were further covered with silica shells (Fig. 17).^70^ Cationic PDDA has roles connection between cells and silica layers, and PSS has another role of protection against ultraviolet light irradiation. The optimized layering of the cells exhibited strong protection effects for very harmful UV-C light irradiation while cell viability and adsorption capability for heavy metal ions of the cyanobacterial cells were preserved. Long-term viability with functions for copper and lead sequestration were actually confirmed. Nanoarchitectonics efforts on the living cells make the cells more useful in applications in harsh conditions.

**Fig. 18:**
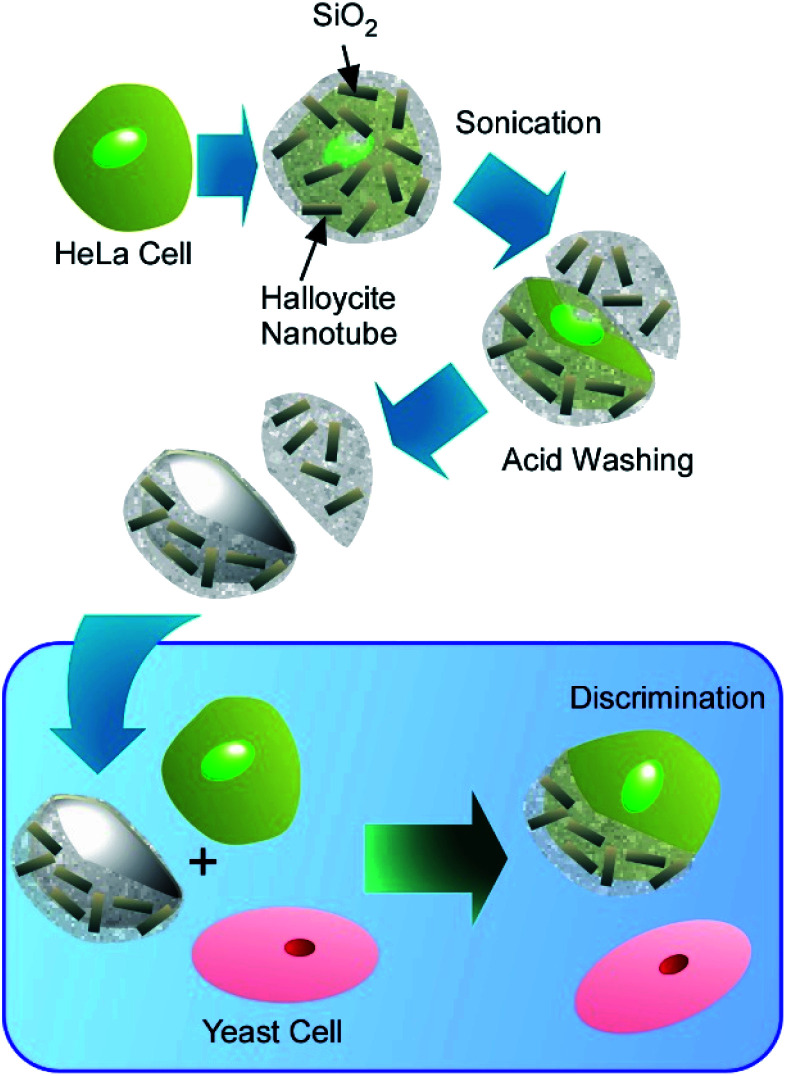
Fabrication of artificial antibodies capable of recognizing specific cells in a cell mixture through cell-surface nanoarchitectonics using cells as sacrificial templates.

The use of cells as sacrificial templates may find application in creation of artificial antibodies capable of recognizing specific cells in a cell mixture (Fig. 18).^71^ The unique surface features of the sacrificed cell become imprinted in the hard shell obtained after the cell template decomposition, enabling it to selectively bind to the similar cells in a cell population. Cell-recognizing silica imprints capable of the selective detection of human cells in a mixture with yeast cells were obtained by using HeLa cells as a template. The silica-halloysite nanotubes shells were fabricated on individual HeLa cells, modified with poly(acrylamide-*co*-diallyldimethylammonium chloride), by biosilification process, followed by incubation with halloysite nanotubes. The success of this early research gives hope that further development of this technique will allow selective recognition of diseased human cells on the basis of changes in the architecture of the cell surface induced by viral infections and neoplastic transformations.

As shown in these examples, decoration of living cells with various inorganic substances is possible, which is sometimes supported with pre-polymer coating. Not limited to cell protection functions, advanced functions of imprinting of cell structures for artificial antibodies were also demonstrated.

## 7. MOF nanoarchitectonics on living cells

Metal–organic frameworks (MOFs),^72^ coordination polymers,^73^ and related regularly structured materials^74^ have been used in various applications because these materials with well-designed components and regular structures can assembled from simple molecular units and metal ions. These materials have been also used as active components of nanoarchitectonics approaches. For example, Liang *et al.* reported biomimetic mineralization of MOF structures on living cells. Crystalline ZIF-8 exoskeleton was formed on surfaces of living yeast cells and bacteria under physiological conditions.^75^ Especially, cell-surface-rich biomolecules such as peptidoglycans and glycoproteins would concentrate precursors of MOF and promote MOF formation. Molecular traffic between external environments and cell inside were regulated by the MOF coating. Cell divisions were prohibited and the cells behaved like those in artificial hibernation conditions. However, removal of the MOF covers from cell surface regenerate full function of the cells. MOF-based deactivation and activation mechanism would have a new type of contributions to single cell technology for biomedical applications.

**Fig. 19:**
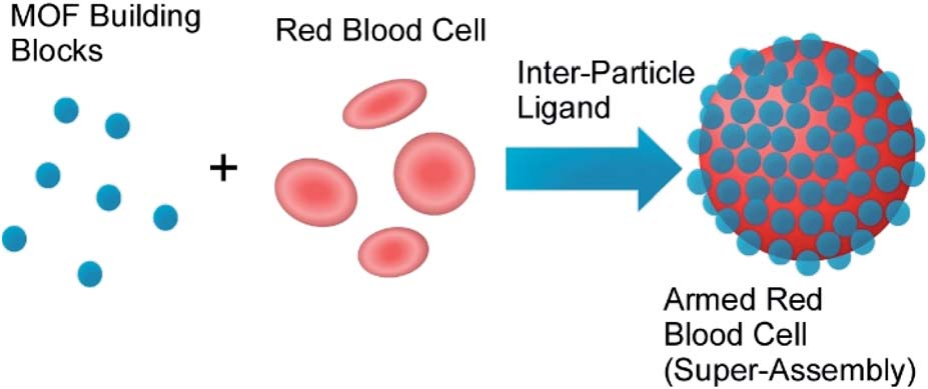
Encapsulation of red blood cells with exoskeleton structures of MOF nanoparticles based on metal–phenolic chemistry.

Zhu, Wuttke, Brinker, and co-workers developed armoured red blood cells through assembling MOF nanoparticles on surface of red blood cells (Fig. 19).^76^ Based on metal–phenolic chemistry, red blood cells were encapsulated by exoskeleton structures of MOF nanoparticles in seconds without lysis of red blood cells, where MOF nanoparticles were interlocked by metal–phenolic coordination and cell membranes and MOF nanoparticles were complexed through hydrogen bonding. Intrinsic functions of red blood cells such as oxygen carrier ability and good circulation capability were maintained even after armoured nanoarchitectonics with MOF nanoparticles. In addition, their resistances against harsh factors including osmotic pressure, freezing environments, toxic substances, and detergents were much enhanced. Furthermore, additional properties such as sensing of blood nitric oxide and multimodal imaging were also realized through modification of physicochemical properties of the MOF nanoparticles.

**Fig. 20:**
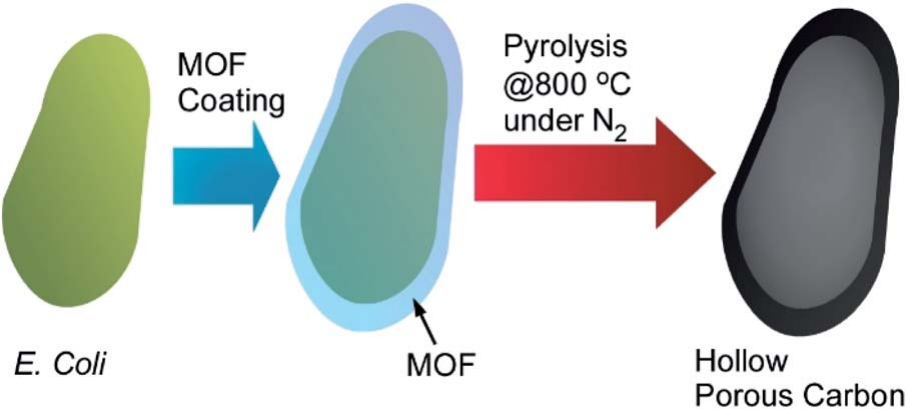
Preparation of hierarchical porous carbon materials from MOF-coated bacterial cells.

Living cells and their derivatized structures are used as templates of MOF-based materials synthesis. Gassensmith and co-workers reported preparation of hierarchical porous carbon materials from MOF-coated bacterial cells (Fig. 20).^77^ The bacterial templates facilitated MOF nanocrystal formation and provided opportunities for morphology and porosity controls of the resulting carbon materials. *Escherichia coli* cells were first subjected as templates for shell formation of zeolitic imidazolate framework-8 (ZIF-8) as a MOF component. Crystal growth of ZIF-8 was somehow disturbed at the *E. coli* cell surface to provide hierarchical structures with micropores, mesopores, and macropores. This pore hierarchy was preserved in the final carbon materials that were obtained after carbonization of MOF-covered *E. coli* cells. This structural characteristic worked advantageously on their electrochemical properties such as specific capacity that was superior to conventional porous carbon materials synthesized from pristine ZIF-8.

**Fig. 21:**
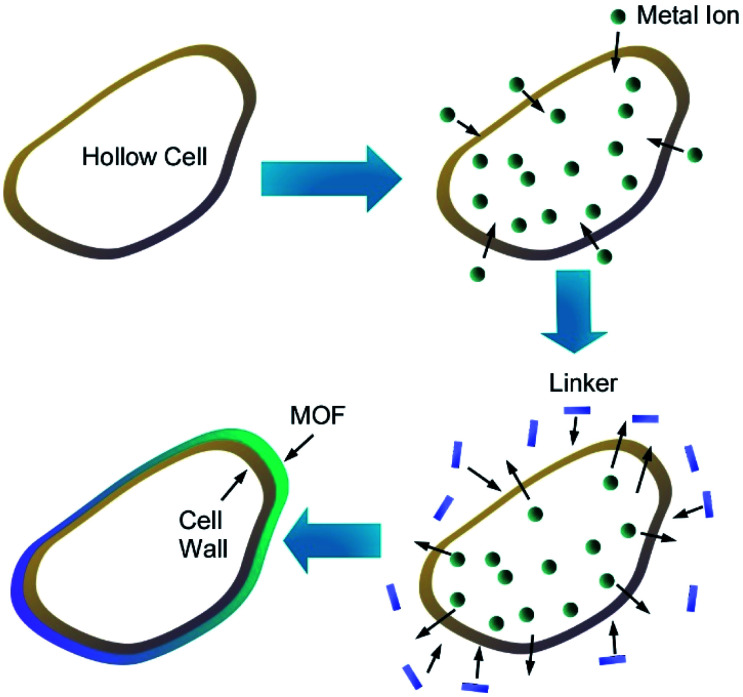
MOF microcapsules with size-selective permeability prepared using cell walls as templates.

Zhang and co-workers prepared MOF microcapsules with size-selective permeability using cell walls as templates (Fig. 21).^78^ Yeast cells were first washed with hot water and methanol several times to provide hollow cell walls upon removal of cytoplasm. Metal ions were then introduced into the resulting hollow cell walls, and addition of precursor ligands from external environments provided opportunity of MOF crystallization at the surface of the cell walls. Based on advantageous features of the cell wall templates including hollow motifs, small-sized pores, and sufficient heterogeneous nucleation sites, uniform MOF layers were grown to nanoarchitect MOF/cell wall microcapsules. In addition to their highly stable nature, controlled release of small molecules with size selectivity was realized through MOF/cell wall membrane. The size selectivity can be adjusted upon selection of deposited MOF types.

**Fig. 22:**
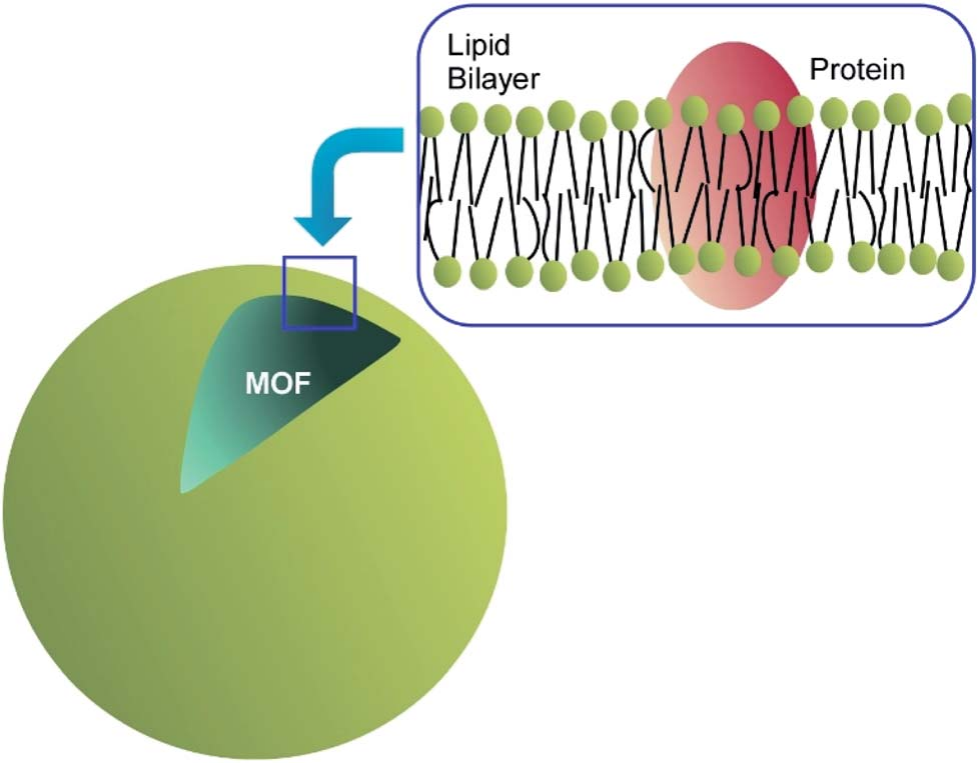
Cell membrane-coated MOF material.

Not limited to living cells, hybridization between biocomponents and MOF materials have been researched. For example, Sumby, Falcaro, Doonan, and co-workers demonstrated enzyme encapsulation by MOF.^79^ Surprisingly, activities of the enzymes have significant dependences on hydrophilicity/hydrophobicity of MOF capsules. Activities of catalase and urease were significantly enhanced in hydrophilic MAF-7 while encapsulation into hydrophobic ZIF-8 resulted in negligible activity for urease and complete deactivation for catalase. As reversed encapsulation nanoarchitectonics, Fang, Zhang, and co-workers fabricated cell membrane-coated MOF materials (Fig. 22).^80^ Cores of the used MOF can stably entrap enzymes, and further coating of the MOF with natural cell membrane led to successful enzyme delivery. It would be useful for enzyme-based therapies for human health conditions. Wei, Luo, Xiao, and co-workers demonstrated multi-functionalized MOF nanoarchitectonics of cell membrane-coated MOF with cancer cell targeting and photodynamic therapy capabilities.^81^ The nanoarchitected materials from MnO_2_ nanosheet-coated MOF and cancer cell membrane shell provided functions of production of singlet oxygen for photodynamic therapy, magnetic resonance imaging, and good membrane stability and integrity during cellular endocytosis.

MOF structures possess specific functions both in component uniqueness and precise structural controllability. MOF nanoarchitectonics on living cells is rather new emerging area but has great potentials in advanced functions.

## 8. Summary and short perspectives

**Fig. 23:**
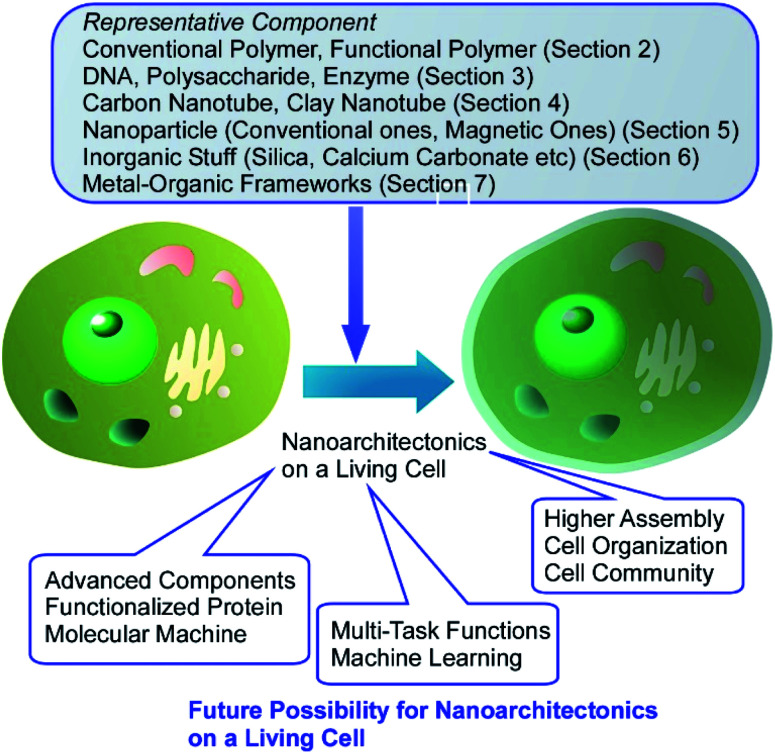
Summary and future perspectives on nanoarchitectonics on living cells.

In this review article, various types of nanoarchitectonics approaches on living cells are discussed. Summary and future perspectives are depicted in Fig. 23. As seen the described examples, we can introduce functional structures with various components on the surface of living cells as if they were immobilized on surfaces of the conventional materials. As shown in this paper, a wide variety of structures materials can be anchored on living cells, ranging from soft components such as organic polymers and biomolecules to non-flexible nanomaterials such as nanoparticles and nanotubes. Coating of living cells with abundant inorganic stuffs and related hybrids are highly possible. The use of advanced structures such as MOF as cell-coating components provides many possibilities in cell regulations. Despite these artificial processes, the cells can remain active or remain in hibernation without being killed. In most the cases, basic functions of the cells are preserved and their resistances against external assaults are much enhanced. Cell-covering with well-designed MOF structures resulted in controls of cell states between normally active and suspended activity through regulations of chemical traffics between cell insides and external environments. The possibilities of nanoarchitectonics on living cells would be high equally to functional modifications of conventional materials. Living cells can be used in common materials sciences.

More advanced surface modifications and the introduction of active objects to the surface of the living cell would add further functionalities to living cells. For example, versatile modifications of membrane proteins would provide new functional opportunities to living cells through protein functions. Recently, Ojida and co-workers reported some useful ways of specific covalent labelling of proteins through interaction between CysHis tag and Ni(ii)-probe pairs.^82^ Similar molecular modification methods would contribute more and more to nanoarchitectonics on living cells. As forefront components, molecular motors were introduced on living cells as demonstrated by García-López *et al.*^83^ Molecular motors introduced in living cells can drill and make holes through living cell membranes. Methodologies for regulations of molecular machines at membrane media have been also proposed.^84^ Interactions and attacks of (inorganic) micromotors to living cells have been similarly demonstrated.^85^ Introduction of these active components will enhance possibilities of dynamic functions in nanoarchitectonics on living cells.

The achieved research efforts and emerging approaches indicate huge possibilities in component selections for living cell decorations. Therefore, designs of multi-task functions with multiple functional units on living cells would integrate cell functions and artificial functions in multiple function links. As seen in successful contributions of artificial intelligence (machine learning) to materials science,^86^ selection and combinations of functional units for nanoarchitectonics on living cells can be optimized with machine learning for specific targets in future. For applicational progresses, further developed organization of decorated cells is necessary. Beyond single cell technology, it may be possible to integrate not just one cell, but a community of cells. In combination with existing cell sheet technologies,^87^ nanoarchitectonics may lead to new functional structures that artificially assemble living organisms. Living cells can be regarded as highly functionalized objects and have indispensable contributions to future materials nanoarchitectonics. Current challenges on nanoarchitectonics on living cells would be establishment of methodology to organize living cells like conventional materials to construct artificial tissue-like structures, which would give high impact to various application fields including tissue engineering and regenerative medicine.

## Author contributions

Both the authors contributed to prepare entire parts of this review. Main contribution of KA is nanoarchitectonics strategies, and living cell descriptions are mainly supported by RF.

## Conflicts of interest

There are no conflicts to declare.

## Supplementary Material
